# Construction of poly(ethylene glycol)-poly(L-lactic acid)-stearic acid reverse aspirin-loaded micelles and optimization of preparation process

**DOI:** 10.1080/15685551.2020.1845428

**Published:** 2020-11-23

**Authors:** Yunpeng Min, Hang Zhang, Huiru Wang, Yimin Song

**Affiliations:** Department of Pharmaceutical Engineering, Qingdao University of Science and Technology, Qingdao, P.R. China

**Keywords:** Reverse micelles, PEG-PLA-SA, aspirin, drug delivery, process Optimization

## Abstract

This work aims to study the construction of reverse aspirin-loaded micelles prepared from amphiphilic PEG-PLA-SA triblock copolymers and the optimization of the preparation process. Using polyethylene glycol (PEG) as the initiator, ring-opening polymerization of L-lactide (L-LA) was used to prepare PEG-PLA diblock copolymers. Final product PEG-PLA-SA triblock copolymers were prepared by the reaction of stearic acid (SA) and PEG-PLA catalyzed by 4-dimethylaminopyridine (DMAP) and N,N’-Dicyclohexylcarbodiimide (DCC). Fourier transform infrared spectrometer (FT-IR) was used to characterize the product structure. PEG-PLA-SA triblock copolymers self-assembled in toluene/ethanol/water system to form reverse micelles, which could encapsulate aspirin into a hydrophilic core. Dynamic light scattering (DLS) and transmission electron microscopy (TEM) were used to determine the size and morphology of reverse micelles. The results showed that the reverse micelles are spherical, with a particle size of less than 70 nm. Response surface analysis method was applied to optimize the preparation process of PEG-PLA-SA. In vitro drug release was achieved by embedding reverse aspirin-loaded micelles in the biocompatible membrane in phosphate buffer saline (PBS) at 37°C. In the first 8 h, the drug release rate of the triblock copolymers was slower than that of the diblock copolymers. After 8 h, the drug release rate of both tended to be flat. The stability of aspirin-loaded reverse micelles was studied through accelerated test. These results indicate that reverse micelle PEG-PLA-SA may be a promising carrier for hydrophilic drugs like aspirin.

## 1. Introduction

Acetylsalicylic acid, or aspirin, first appeared as an analgesic in the form of willow bark, and its antipyretic and analgesic effects have been recognized for more than 200 years. Later, the antiplatelet activity of aspirin was discovered, which prompted us to further study the mechanism of action of aspirin and the clinical effect of this drug in the treatment of common cardiovascular diseases [1–3]. Aspirin is a commonly used antiplatelet drug that can reduce the risk of recurrence or cancer-specific mortality [4,5]. Long-term prophylactic use of low-dose aspirin reduced the incidence of heart attacks and strokes in cancer, and the benefits of aspirin for cancer did not appear until three years after the drug was started, and for long-term aspirin users, the benefits lasted several years after discontinuation [6–9]. Platelet activation has been investigated for its role in inflammatory response by inducing, aggregating and activating white blood cells and other platelets, and aspirin has been shown to modulate various pathogenic mechanisms of multifunctional dysfunction in sepsis and ARDS, as well as a potential treatment for sepsis and acute respiratory distress syndrome (ARDS) [10]. Although neurodegenerative diseases such as stroke, Alzheimer’s and Parkinson’s show different pathologies, inflammation and oxidative stress are common causes, and the anti-inflammatory substance aspirin may have a potential neuroprotective effect in these diseases [11]. Because oral low-dose aspirin can also cause side effects in the gastrointestinal tract, the filtration of metabolism requires frequent administration, and a lower utilization of the drug will cause additional metabolic burdens. Therefore, it is very important to find a more convenient and safer carrier for aspirin delivery, especially during long-term use [12].

During the past few decades, polymeric micelles have raised special attention as novel nano-sized drug delivery systems for optimizing the treatment and diagnosis of numerous diseases [13]. These nanocarriers exhibit several in vitro and in vivo advantages as well as increased stability with delivery of hydrophilic drugs controlled by micelle formation [14]. In addition, mixed polymer micelles were proven to be better drug delivery systems than simple polymer micelles [15]. PEG-PLA is one of the most prominent amphiphilic polymers and therefore very suitable for micelles. PLA is a biodegradable and biocompatible polyester in the form of a renewable resource and has been approved by the US Food and Drug Administration (FDA) for clinical use [16,17]. The hydrophobicity of PLA makes it suitable for the hydrophobic part of micelles. Among them, PEG is the most popular hydrophilic agent due to its various advantages, including linearity, undercharge, immunogenicity, low polydispersity and easy coupling activation. PEG-PLA micelles have been widely used in drug delivery systems duo to their excellent physicochemical and biological properties, non-toxic, non-protein adsorption and reduced absorption of reticuloendothelial system (RES) after intravenous injection [18,19]. Since the low encapsulation rate of PEG-PLA for aspirin remains to be solved, various modifications have been made to the profile of PEG-PLA, and the synthesis process has been optimized. An endogenous long-chain saturated fatty acid has been selected, which is widely used in pharmaceutical. Stearic acid is the main component of fat, which has the characteristics of good biocompatibility and low cyotoxicity [20]. So far, the preparation of micelles is usually done by emulsion-solvent evaporation or emulsion-solvent diffusion, but these techniques are only suitable for encapsulating hydrophobic drugs [21]. Such forward micelles that can dissolve nonpolar compounds have been extensively studied. Conversely, there is little literature on reverse micelles that can dissolve polar compounds.

The reverse micelle of PEG-PLA-SA could be prepared by self-assembly of solvent/cosolvent/water system. Toluene was used as solvent and ethanol as co-solvent to make the nano-scale reverse micelle produced by dissolving appropriate amount of water capable of this. Aspirin was wrapped in the hydrophilic core to construct ASA/PEG-PLA-SA reverse drug-loaded micelles. Reverse aspirin-loaded micelles were embedded into the biocompatible membrane for in vitro drug release study to evaluate the potential application of PEG-PLA-SA as a aspirin medical carrier.

## 2. Experimental

### 2.1. Materials

L-lactide were obtained from Aladdin Reagent Company and purified by crystallization from ethyl acetate. Monomethoxy poly(ethylene glycol) (mPEG) with molar masses of 2000 to 8000, stearic acid, aspirin, N,N’-Dicyclohexylcarbodiimide (DCC), 4-dimethylaminopyridine (DMAP) and zinc lactate were purchased from Sinopharm Chemical Reagent Company. All organic solvents were of analytic grade and used without further purification.

### 2.2. Methods

#### 2.2.1. Synthesis of PEG-PLA copolymers

The PEG-PLA diblock copolymers were synthesized by ring-opening polymerization. Predetermined amounts of PEG and L-lactide were introduced into a dry three-necked flask, with an initial molar ratio of ethylene oxide to lactyl repeat units (EO/LA) ranging from 1 to 8, zinc lactate (0.1 wt%) added then. Vacuum and nitrogen filling were repeated 3–4 times. The reaction was carried out at 140°C for 24 h under vacuum. After cooling down, the product was dissolved in anhydrous dichloromethane and precipitated in anhydrous ether. After suction filtration, PEG-PLA diblock copolymers were obtained and dried under vacuum to constant weight.

#### 2.2.2. Synthesis of PEG-PLA-SA copolymers

Dissolved 2 mmol of SA and 2 mmol of PEG-PLA diblock copolymers in dichloromethane, then added 0.2 mmol of DMAP and 2 mmol of DCC sequentially. Nitrogen protection, stirring, reaction at 30°C for 36 h were performed followed by filtering to remove the sink, then the solvent was distilled off under reduced pressure, and then dissolved in ethyl acetate, extracted with 300 g/L NaCl aqueous solution multiple times to wash away residual PEG-PLA. After adding anhydrous MgSO_4_, the solvent ethyl acetate was distilled off under reduced pressure and dried under vacuum at 40°C for 24 h to obtain a white solid product.

### 2.3. Preparation of blank and ASA-loaded reverse micelles

For the preparation of blank reverse micelles, 1 g of PEG-PLA diblock and 1 g of PEG-PLA-SA triblock copolymers were dissolved in 20 mL of a mixture of toluene-ethanol (3:1) respectively. 0.5 mL of distilled water was then added to each copolymer solution [22]. The mixture was homogenized by vortex for 10 min to yield a transparent or translucent solution. The same procedure was used to prepare aspirin-loaded reverse micelles, except that distilled water was replaced by 2.5 mL aspirin solution (4 mg/mL).

### 2.4. Determination of entrapment efficiency and drug loading

Encapsulated levels of aspirin were measured using UV spectrophotometer. Lyophilized micelles were dissolved in the acetonitrile to disrupt the self-assembled structures. This solution was subjected to UV spectrophotometer Shimadzu 2450, Shimadzu Corporation (Kyoto, Japan) analysis of aspirin at 242 nm.

### 2.5. In vitro release of ASA from membranes with embedded aspirin-loaded reverse micelles

The aspirin-loaded reverse micelles solution was prepared by mixing 1 g copolymer in different proportions and 2.5 mL aspirin solution (4 mg/mL) in 10 mL toluene-ethanol (3:1) solution. Membranes were prepared by mixing the aspirin-loaded reverse micelles solution with a solution 10 % PLA in toluene with a volume ratio of 1:3. The organic phase was then removed by reduced pressure rotary evaporation. Residual solvents were thoroughly eliminated through vacuum drying. Aspirin release from polymeric membranes with embedded reverse micelles was performed in pH 7.4 phosphate-buffered saline (PBS) at 37°C. Each membrane was placed in 10 mL PBS. At give time intervals, 0.5 mL aliquots of the release medium were withdrawn and replaced by 0.5 mL of fresh PBS to maintain the sink condition.

### 2.6. The stability analysis of aspirin-loaded reverse micelles

Place the dried aspirin-loaded PEG-PLA-SA reverse micelles (The preparation method of aspirin-loaded reverse micelles was the same as 2.5) in a constant temperature and humidity chamber with a relative temperature of 40 °C (±2) and humidity of 75% (±5) a month. The stability of micelles was determined by in vitro release profile of the actual content of aspirin per gram of polymer in 24 h. Samples were tested at the beginning, middle and end of the month three times in parallel.

### 2.7. Characterizations

#### 2.7.1. Fourier transform infrared spectrometer(FT-IR)

An appropriate amount of PEG-PLA, SA and PEG-PLA-SA were respectively mixed with 100 mg KBr as the samples, and the thin films were prepared by pressing method. Then, the samples were analyzed by Bruker tensor-27 Fourier Transform Infrared Spectrometer (TENSOR, Germany) at wavelengths in the range of 400–4000 cm^−1^.

#### 2.7.2. Dynamic light scattering(DLS)

The mean size of the polymeric reverse micelles was analyzed by DLS using photon Cross-correlation Spectroscopy (Nanophox, Sympatec). Quartz cuvettes were used for all DLS tests which were carried out at 25°C.

#### 2.7.3. Transmission electron microscopy(TEM)

The morphology of blank and aspirin-loaded reverse micelles was observed by TEM using a JEOL 1200 EXII instrument operating at an acceleration voltage of 120 kV. Five microliters reverse micelles solution was dropped onto a copper grid covered with collodion-carbon, and air-dried at room temperature before measurements.

#### 2.7.4. High-performance liquid chromatography(HPLC)

The following chromatographic conditions were used to determine the concentration of aspirin by high-performance liquid chromatography. The chromatographic column adopts InertsilODS-SP. The column temperature was set at 30°C with 10 μL sample loaded. The mobile phase is water-methanol (3:7). The flow rate is 0.5 mL/min, and the detection wavelength is 280 nm.

## 3. Results and discussion

**Figure 1.**
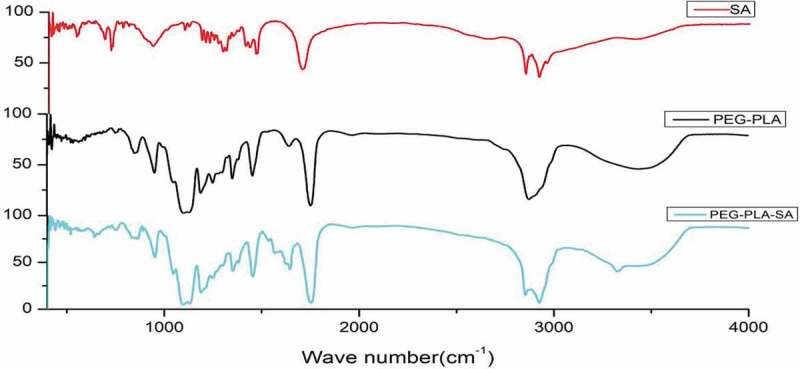
FT-IR of SA, PEG-PLA diblock and PEG-PLA-SA triblock copolymers

### 3.1. Synthesis and characterization of PEG-PLA diblock and PEG-PLA-SA triblock copolymers

PEG-PLA-SA triblock polymer was synthesized by two-step reaction ring-opening polymerization and esterification reaction. Monomethoxy PEG (mPEG) with Mn of 2000,3000,4000,5000 and 8000 was used in this work, yielding PEG-PLA diblock and PEG-PLA-SA triblock copolymers as shown in Table 1. summaried the molecular characteristics of the various diblock and triblock copolymers. For the sake of clarity, triblock copolymers were named as EO_X_LA_Y_SA, and diblock copolymers as EO_X_LA_Y_. In these acronyms, SA, LA and EO represented stearic acid, L-PLA and PEG blocks, respectively, x and y representing the number-average degree of polymerization of corresponding blocks. As shown in Table 1, DP_PEG_ ranged from 45 for mPEG2000 to 182 for mPEG8000, while DP_PLA_ ranged from 6 for diblock or triblock EO_45_LA_6_(SA) to 42 for diblock or triblock EO_182_LA_42_(SA). The Mn of the copolymers varied from 2430 for EO_45_LA_6_ to 11,305 for EO_182_LA_42_SA. The connection between the three substances could be determined by FT-IR. As shown in Figure 1, from the infrared spectrum of PEG-PLA-SA, it could be seen that at 1735.21 cm^−1^, the ester bond absorption peak compared to PEG-PLA was enhanced, which explained the formation of ester bond between PLA and SA. While the hydroxyl absorption peak of PEG-PLA-SA weakened, it meant the hydroxyl on the PLA was bound by SA. It showed that the polymer PEG-PLA-SA was successfully synthesized.

**Table 1.** Molecular characteristics of PEG-PLA and PEG-PLA-SA copolymers

Run	Acronym	*M*_nPEG_(g/mol)	*DP*_PEG_	*DP*_PLA_	*DP*_PEG_/*DP*_PLA_	*M*n
1	EO_45_LA_6_	2000	45	6	7.50	2430
2	EO_45_LA_18_	2000	45	18	2.50	3300
3	EO_45_LA_31_	2000	45	31	1.45	4230
4	EO_68_LA_42_	3000	68	42	2.83	6020
5	EO_113_LA_42_	5000	113	42	2.69	8020
6	EO_168_LA_42_	8000	182	42	4.33	11,020
7	EO_45_LA_6_SA	2000	45	6	7.50	2715
8	EO_45_LA_18_SA	2000	45	18	2.50	3585
9	EO_45_LA_31_SA	2000	45	31	1.45	4515
10	EO_68_LA_42_SA	3000	68	42	2.83	6305
11	EO_113_LA_42_SA	5000	113	42	2.69	8305
12	EO_168_LA_42_SA	8000	182	42	4.33	11,305

### 3.2. Preparation and characterization of blank and ASA-loaded reverse micelles

Reverse micelles were prepared by dissolving copolymers in a toluene-ethanol mixture, followed by addition of distilled water and homogenization. DLS was used to determine the size of reverse micelles. Table 2 summarizes the mean size of various copolymer micelles. The average diameter of reverse micelles ranged from 20 nm to 70 nm. It can be seen from that the particle size of the aspirin-loaded micelles is larger than that of the blank micelles with the same block ratio. It is worth noting that triblock copolymers exhibited smaller micelle size than diblock copolymers.

**Table 2.** Characteristics of blank and aspirin-loaded reverse micelles of PEG-PLA and PEG-PLA-SA determined by DLS

copolymer	Mean size(nm) (blank)	Mean size(nm) (aspirin-loaded)
EO_45_LA_6_	22.5	25.6
EO_45_LA_18_	35.3	48.4
EO_45_LA_31_	46.9	47.3
EO_68_LA_42_	50.2	58.2
EO_113_LA_42_	61.2	61.6
EO_182_LA_42_	57.3	59.8
EO_45_LA_6_SA	18.7	30.1
EO_45_LA_18_SA	36.8	39.5
EO_45_LA_31_SA	45.1	45.5
EO_68_LA_42_SA	42.6	49.8
EO_113_LA_42_SA	60.6	61.2
EO_182_LA_42_SA	56.9	69.6

**Figure 2.**
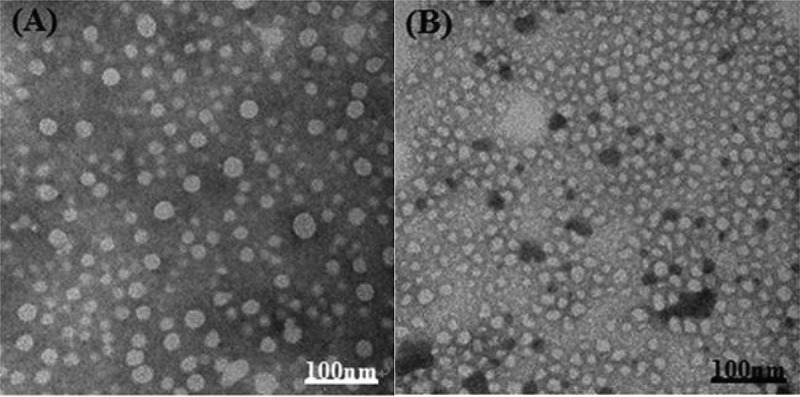
TEM photographs of aspirin-loaded reverse micelles(a) and blank reverse micelles (b) of EO_45_LA_18_SA copolymer

The morphology of reverse micelles was examined under TEM as shown in Figure 2. The micelles were spherical in shape and exhibited mean diameters slightly smaller than the values observed by DLS. On the other hand, the mean size of aspirin-loaded reverse micelles was obviously larger than that of blank ones.

### 3.3. Determination of entrapment efficiency and drug loading

The levels of encapsulated aspirin were measured using UV detection. Lyophilized micelles were dissolved in theacetonitrileto disrupt the self-assembled structures. This solution was then subjected to UV spectrophotometer Shimadzu 2450, Shimadzu Corporation (Kyoto, Japan) for analysis of aspirin at 242 nm. Aspirin concentration was determined by drug substance calibration curve. Polymer concentration was calculated from the initially used polymer feed prior to the preparation of micelles.

(1) $$LE=\frac{weightofloadeddrug}{theoreticaldrugloading}\times 100\%$$

(2) $$LC=\frac{weightofloadeddrug}{weightofdrug-loadedmicelles}\times 100\%$$

### 3.4. ASA/PEG-PLA-SA process optimization

#### 3.4.1. Design of response surface test

By Selecting the molecular weight of PEG, the molar weight of stearic acid, the amount of L-lactide, the reaction temperature, the reaction time, the molar ratio of the catalyst DCC to DMAP, and the concentration of aspirin for single factor, the amount of L-lactide, the amount of stearic acid, reaction temperature and reaction time were determined with greatest influence on the encapsulation efficiency of drug-loaded micelles. In order to further study the influence of the interaction between these parameters, response surface analysis was used to screen the best extraction process. The preliminary study of each single factor is the guidance range of response surface optimization. Based on the Box-Behnken sampling principle, four factors affecting Entrapment efficiency are selected as follows: Reaction temperature(A), The amount of SA(B), The amount of L-lactide(C), Reaction Time(D) performing a response surface analysis test with four factors and three levels. Response surface test factor levels and results are shown in Tables 3 and 4.

**Table 3.** Levels and codes of response surface test factors

coding	Reaction temperature A/(°C)	The amount of SA B/(mol)	The amount of L-lactide C/(mol)	Reaction time D/(h)
−1	25	0.01	0.03	30
0	30	0.03	0.06	36
1	35	0.05	0.09	42

**Table 4.** Experimental design and results

Serial number	Reaction temperature A/(°C)	The amount of SA B/(mol)	The amount of L-lactide C/(mol)	Reaction time D/(h)	Entrapment efficiency/(%)
1	−1	0	−1	0	11.1
2	−1	1	0	0	24.9
3	1	0	1	0	38.7
4	0	0	1	1	54.8
5	1	1	0	0	45.7
6	0	−1	0	−1	40.2
7	0	0	−1	1	37.5
8	0	0	0	0	67.6
9	0	1	1	0	59.1
10	0	−1	1	0	47.4
11	0	−1	0	1	57.9
12	0	0	0	0	65.1
13	0	1	0	−1	47.3
14	0	0	0	0	63.8
15	−1	0	1	0	27.9
16	0	0	0	0	62
17	0	0	0	0	60.5
18	−1	0	0	−1	18.7
19	−1	0	0	1	24
20	0	−1	−1	0	33.5
21	−1	−1	0	0	26.8
22	1	0	−1	0	13.2
23	0	0	1	−1	50.4
24	0	1	−1	0	36.8
25	0	0	−1	−1	32.9
26	1	0	0	−1	25.3
27	1	−1	0	0	26.4
28	1	0	0	1	32.8
29	0	1	0	1	50.9

#### 3.4.2. Response surface test results and analysis of variance

Use Design-Expert 10.0.3 software to analyze the experimental data and get the quadratic polynomial regression equation:

Entrapment efficiency (%) = 63.8 + 4.05833 * A + 2.70833 * B + 9.44167 * C + 3.59167 * D + 5.3 * AB + 2.175 * AC + 0.55 * AD + 2.1 * BC -3.525 * BD -0.05 * CD -28.4708 * A^2^ -5.79583 * B^2^ -12.4958 * C^2^ -8.82083 * D^2^ (encoding system). The analysis of variance on the regression equation is shown in Table 5. Among them, the F value can be used to test the significance of the impact of each variable on the response value. The larger the F value, the higher the significance of the corresponding variable. When the model’s significance test probability P < 0.05, the model is considered to be statistically significant. It can be seen from Table 5 that the order of the influence of process conditions on entrapment efficiency is: C > A > D > B, that is, The amount of L-lactide> Reaction temperature> Reaction time> The amount of SA. The coefficient of determination R^2^ of the model is 0.9814, indicating that the model is highly significant. At the same time, R^2^adj = 0.9629 can explain 96.29% of the experimental response variation, and it is also close to the predicted correlation coefficient Pred R^2^, indicating the degree of fitting between the experimental model and the real data, with practical guiding significance, so the model can be used to analyze and predict the optimal extraction process for entrapment efficiency.

**Table 5.** Results of analysis of variance fitting regression equation

Source of Variance	Sum of squares	Degree of freedom	Variance	F value	P value	Significance
Regression model	7352.14	14	525.15	52.86	< 0.0001	**
A	197.64	1	197.64	19.89	0.0005	**
B	88.02	1	88.02	8.86	0.0100	*
C	1069.74	1	1069.74	107.67	< 0.0001	**
D	154.80	1	154.80	15.58	0.0015	**
AB	112.36	1	112.36	11.31	0.0046	**
AC	18.92	1	18.92	1.90	0.1892	
AD	1.21	1	1.21	0.12	0.7323	
BC	17.64	1	17.64	1.78	0.2040	
BD	49.70	1	49.70	5.00	0.0421	
CD	1.000E-002	1	1.000E-002	1.006E-003	0.9751	
A^2^	5257.87	1	5257.87	529.19	< 0.0001	**
B^2^	217.89	1	217.89	21.93	0.0004	**
C^2^	1012.84	1	1012.84	101.94	< 0.0001	**
D^2^	504.69	1	504.69	50.80	< 0.0001	**
Residual	139.10	14	9.94			
Lack of fit	108.84	10	10.88	1.44	0.3877	
Pure error	30.26	4	7.56			
Total	7491.24	28				

R^2^ = 0.9814, Adj R^2^=0.9629, Pred R^2^=0.9100

#### 3.4.3. Optimization of entrapment process for response surface analysis of various factors

**Figure 3.**
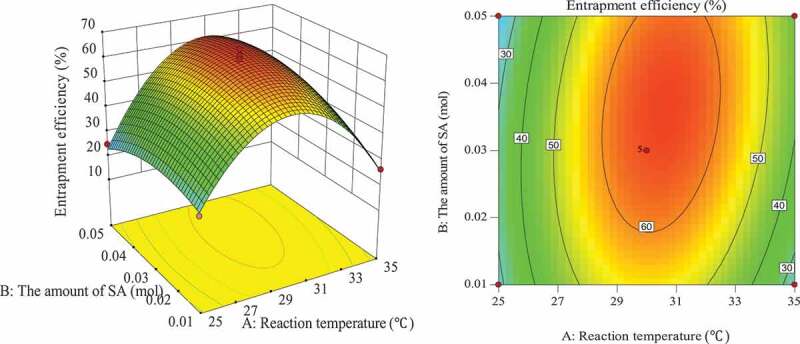
Effect of reaction temperature and the amount of SA on entrapment efficiency

**Figure 4.**
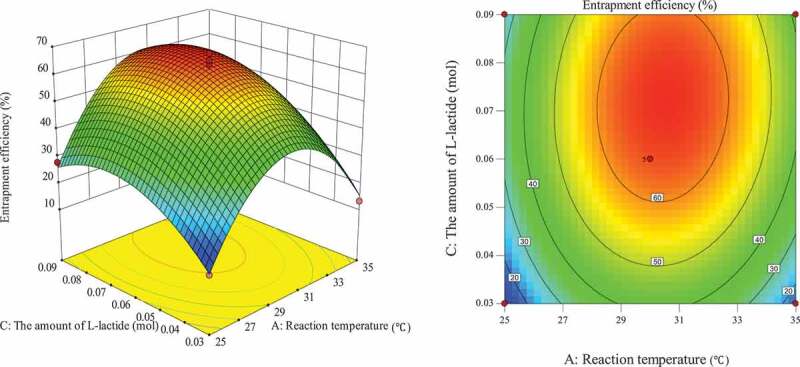
Effect of the amount of L-lactide and reaction temperature on entrapment efficiency

**Figure 5.**
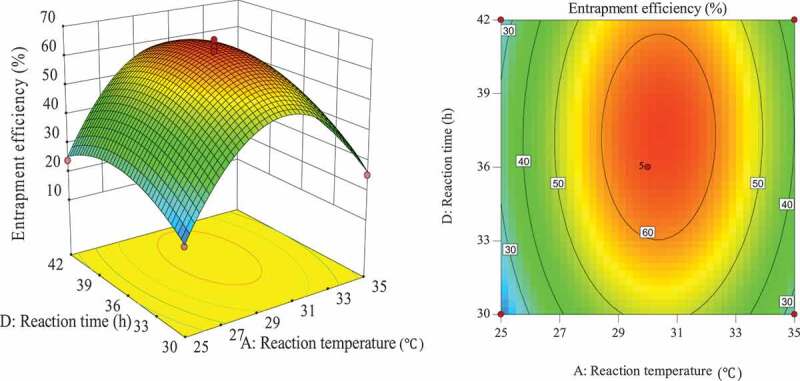
Effect of reaction time and reaction temperature on entrapment efficiency

**Figure 6.**
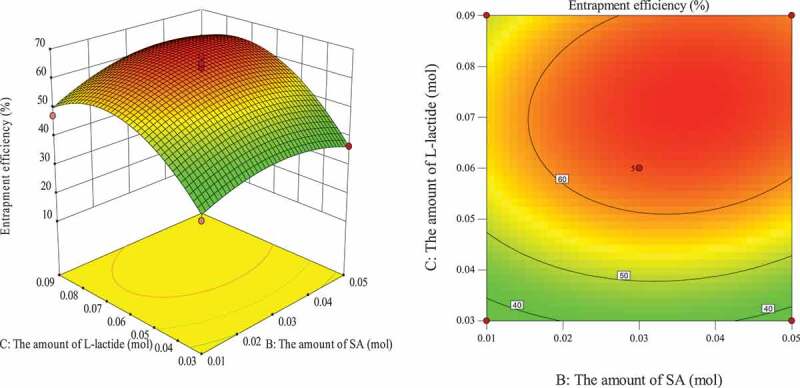
Effect of the amount of SA and the amount of L-lactide on entrapment efficiency

**Figure 7.**
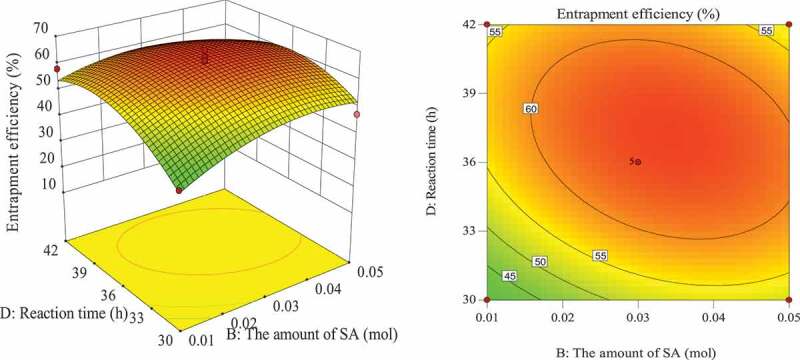
Effect of reaction time and the amount of SA on entrapment efficiency

**Figure 8.**
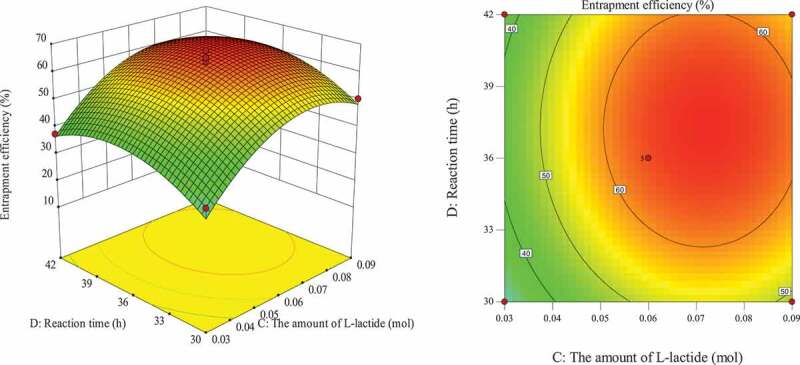
Effect of reaction time and the amount of L-lactide on entrapment efficiency

The influence of the interaction of different process conditions on entrapment efficiency is shown in Figures 3 to 8. It can be seen from Figure 3 that the influence of the reaction temperature-amount of SA interaction on entrapment efficiency is an arched surface. Entrapment efficiency increases firstly and then decreases with the increase of reaction temperature, and it is nearly linear with the increase of the amount of SA. In comparison, the reaction temperature causes greater fluctuations in the surface, indicating that the reaction temperature has a greater contribution to the interaction between them and has a greater impact on entrapment efficiency. When the reaction temperature of the sensitive factor is in the range of 29–31°C, the entrapment efficiency can be improved. As shown in Figure 4, the influence of the interaction between reaction temperature and the amount of L-lactide on entrapment efficiency has a parabolic surface distribution. Entrapment efficiency firstly increases and then decreases with the increase of reaction temperature and the amount of L-lactide. Considering only the entrapment efficiency under the influence of the two factors, the optimized process conditions are the combination of reaction temperature 29–32°C and the amount of L-lactide 0.05–0.09 mol. In the reaction temperature-reaction time interaction shown in Figure 5, the reaction temperature causes a large change in the response surface, indicating that the influence of this factor on entrapment efficiency is more significant than that of reaction time. When the reaction temperature is set to 29–31°C, the entrapment efficiency can be promoted. It can be seen from Figure 6 that the interactive surface shows a trend of inclined surface change, and the magnitude of the change of entrapment efficiency with the amount of L-lactide is greater than the influence of the amount of SA. When the amount of SA is less than 0.03 mol, the entrapment efficiency is positively correlated with the amount of SA; and when the amount of SA is greater than 0.03 mol, the correlation will be inverted. When the amount of SA takes a value near 0.03 mol, it reaches a critical optimum process parameter of entrapment efficiency. Similarly, the critical optimum parameter of the amount of L-lactide is 0.07 g. In the interaction shown in Figure 7, the entrapment efficiency fluctuates more in the direction of reaction time than in the amount of SA, indicating that the former is more sensitive to the impact of entrapment efficiency than later. The influence of reaction time on entrapment efficiency firstly increased and then decreased, while entrapment efficiency increased firstly and then leveled off with the increase of the amount of SA. In order to improve entrapment efficiency, the key factor, reaction time, should be controlled to be greater than 33 h. In the interaction surface as shown in Figure 8, the longitudinal span of the surface in the direction of the amount of L-lactide is larger, indicating that the contribution of the amount of L-lactide in the interaction is greater than reaction time. Taking the combination of the two factors at a moderate level, the amount of L-lactide 0.06–0.09 mol and the reaction time 33–42 h could significantly enhance the entrapment efficiency.

#### 3.4.4. Verification of the optimal process conditions

In order to coordinately consider the influence of the interaction between various factors on entrapment efficiency, and further determine the global optimal solution, with the maximum entrapment efficiency as the optimization goal, according to the results of Design-Expert 10.0.3 software, as in reaction condition as 30.592°C, 0.036 mol of SA, 0.072 mol of L-lactide, and 36.848 h, the entrapment efficiency predicted by the model is 66.686%. According to the software prediction results, combined with the feasibility of the actual process setting, in reaction temperature at 30.6°C, the amount of SA 0.036 mol, the amount of L-lactide 0.072 mol, and the reaction time 36.8 h as conditions to carry out three repeated times, the average entrapment efficiency is 66.89%, which is close to the model prediction result, indicating that the method of optimizing the entrapment efficiency extraction process based on the response surface model analysis is effective and feasible.

### 3.5. In vitro release of aspirin from membranes with embedded aspirin-loaded reverse micelles

**Figure 9.**
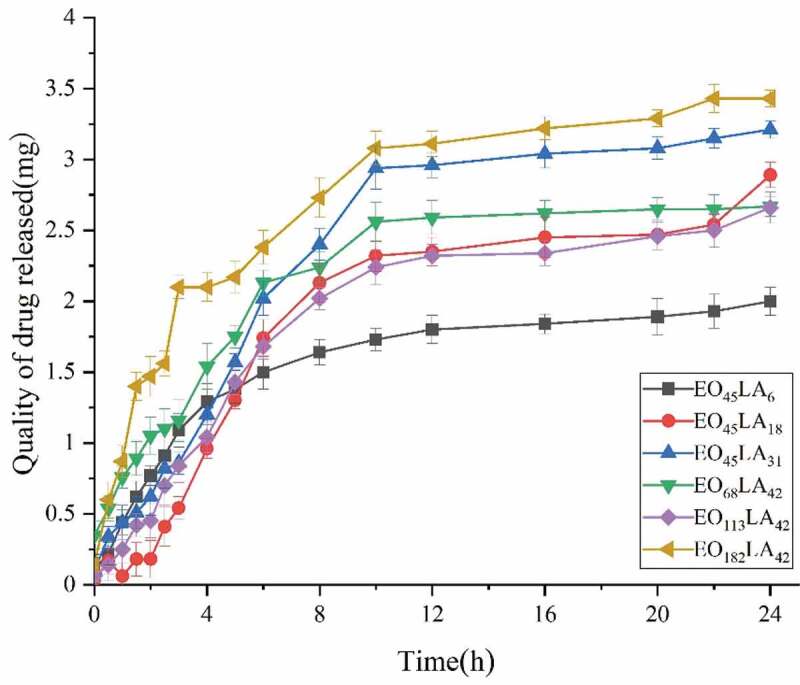
Aspirin release profiles per gram of the copolymer in PH 7.4 PBS at 37 °C using HPLC. (EO_45_LA_6_, EO_45_LA_18_, EO_45_LA_31_, EO_68_LA_42_, EO_113_LA_42_, EO_182_LA_42_)

**Figure 10.**
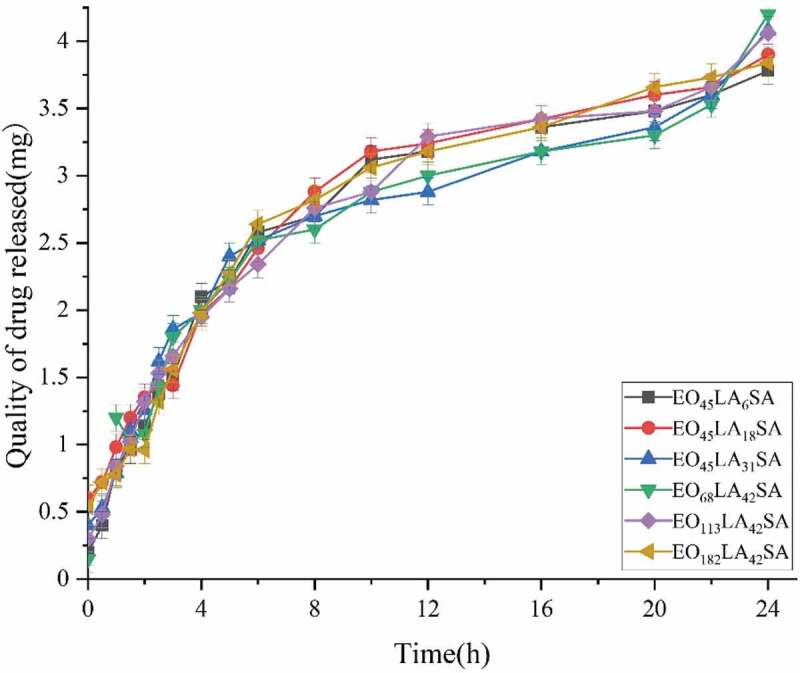
Aspirin release profiles per gram of the copolymer in PH 7.4 PBS at 37 °C using HPLC. (EO_45_LA_6_SA, EO_45_LA_18_SA, EO_45_LA_31_SA, EO_68_LA_42_SA, EO1_13_LA_42_SA, EO_182_LA_42_SA)

The release model of reverse aspirin-loaded micelles embedded in the membrane depends on the ratio of the two solutions. In order to obtain a membrane with good mechanical strength, a ratio of 1:3 of reverse micelles to PLA solutions was selected to prepare membrane controlled release system. According to the aspirin-loaded reverse micelles prepared in **2.5**. By the formula for calculating the encapsulation efficiency, the quality of drug actually loaded into diblock polymers is approximately 3 mg (2.3 mg, 3.4 mg, 3.2 mg, 2.8 mg, 3.5 mg and 2.7 mg for EO_45_LA_6_, EO_45_LA_18_, EO_45_LA_31_, EO_68_LA_42_, EO_113_LA_42_ and EO_182_LA_42_). The quality of drug actually loaded into triblock polymers is approximately 6 mg (5.6 mg, 6.1 mg, 5.8 mg, 6.3 mg, 6.5 mg and 5.7 mg for EO_45_LA_6_SA, EO_45_LA_18_SA, EO_45_LA_31_SA, EO_68_LA_42_SA, EO_113_LA_42_SA and EO_182_LA_42_SA). In vitro release of aspirin in membranes with embedded reverse micelles was achieved in PBS at 37°C, pH 7.4. As shown in Figures 9 and 10, the drug release rate of diblock and triblock polymers is relatively fast in the first 8 h, but compared with triblock polymers, the release rate of diblock polymers is faster. The quality of cumulative drug release of diblock polymers reached 2 mg in the first 8 h except EO_45_LA_6_ with 1.64 mg (2.13 mg, 2.4 mg, 2.24 mg, 2.02 mg, and 2.73 mg for EO_45_LA_31_, EO_45_LA_18_, EO_68_LA_42_, EO_113_LA_42_ and EO_182_LA_42_). The quality of cumulative drug release of triblock polymers not reached 2 mg in the first 8 h except EO_45_LA_18_SA with 2.15 mg (1.75 mg, 1.96 mg, 1.93 mg, 1.72 mg and 1.83 mg for EO_45_LA_6_SA, EO_45_LA_31_SA, EO_68_LA_42_SA, EO_113_LA_42_SA and EO_182_LA_42_SA). The release rates of both decreased after 8 h, almost all drugs loaded in the diblock polymers were released within 24 h (2 mg, 2.89 mg, 3.21 mg, 2.67 mg, 2.66 mg and 3.43 mg for EO_45_LA_6_, EO_45_LA_18_, EO_45_LA_31_, EO_68_LA_42_, EO_113_LA_42_ and EO_182_LA_42_). The cumulative drug release of triblock polymers reached 70% within 24 h (3.78 mg, 3.9 mg, 4.08 mg, 4.2 mg, 4.06 mg and 3.84 mg for EO_45_LA_6_SA, EO_45_LA_31_SA, EO_45_LA_18_SA, EO_68_LA_42_SA, EO_113_LA_42_SA and EO_182_LA_42_SA). As shown in Figures 9 and 10, considering the degree of polymerization of PEG or PLA alone, it has almost no effect on the release rate.

### 3.6. The stability analysis of aspirin-loaded reverse micelles

**Figure 11.**
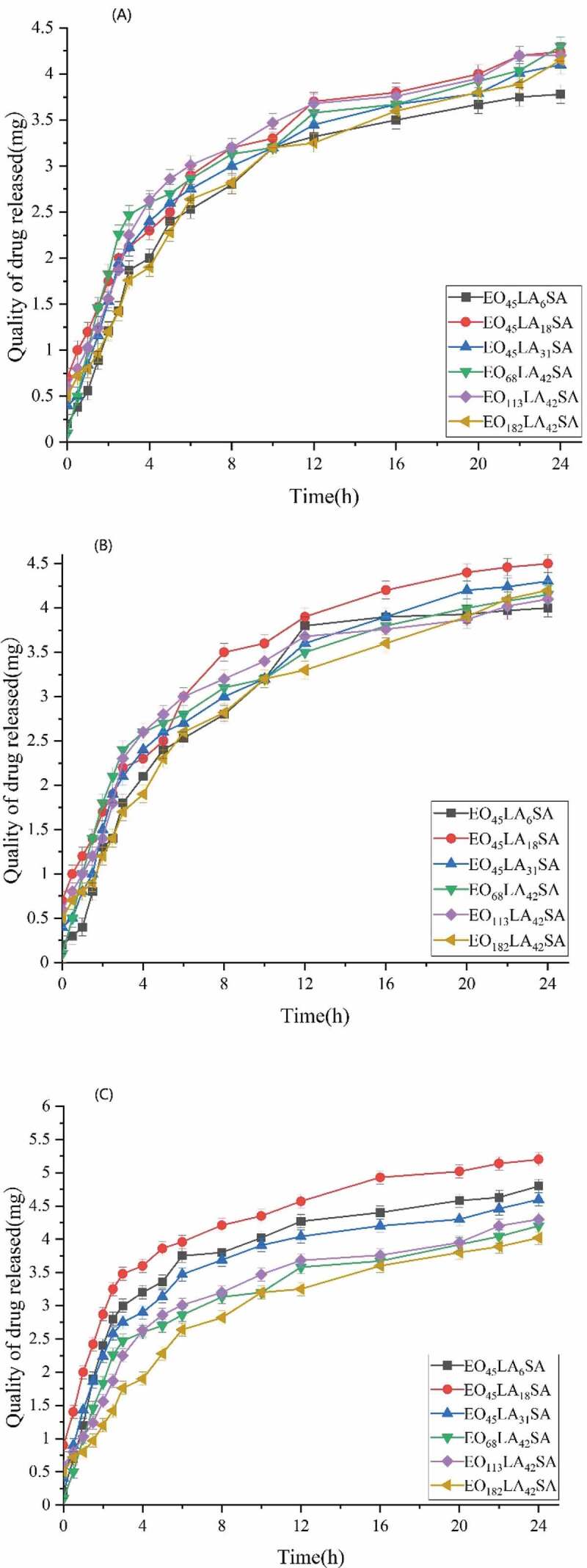
Aspirin release profiles per gram of the copolymer under accelerated conditions at the beginning(a), middle(b) and end(c) of month

Figure 11 shows that the drug release profiles of triblock polymers at different acceleration time. It could be seen from Figure 11 that the cumulative drug release of triblock polymers in 24 hours showed a slight increasing trend, but there was no significant difference. Moreover, the cumulative drug release at different acceleration time was more than 70%. The results showed that aspirin-loaded reverse micelles had good stability

### 3.7. Data analysis

Reverse micelle PEG-PLA-SA is formed by self-assembly in toluene/ethanol/water co-solvent, with PEG segment as the hydrophilic core and PLA-SA as the hydrophobic shell, which can encapsulate aspirin in the hydrophilic core. Such micelles are called reverse micelles, which can encapsulate hydrophilic drugs. According to the literature, when the ratio of toluene to ethanol is 3:1, the reverse micelles prepared show the best transparency, uniformity, solubilization and DLS analysis effect [22]. The reverse micellar solutions used in the experiment were all dissolved in 20 mL of toluene/ethanol (3:1) mixed solution with 2 g copolymer.

We know that the physical and chemical properties of drugs determine the characteristics of the drug delivery system we choose. Due to the good water solubility of aspirin, aspirin is easily degraded during delivery, so reverse micelles with a hydrophilic core are used as the delivery medium of aspirin. In this experiment, the toluene/ethanol/copolymer solution was uniformly stirred to form a stable reverse micelle solution (the ratio of toluene/ethanol was 3:1, and the 10% copolymer ensured the formation of reverse micelle solution), and then the aspirin solution is directly added to it to form a reverse drug-loaded micelle system of ASA/PEG-PLA-SA, with aspirin wrapped in the hydrophilic core of the reverse micelle. According to the DLS results, the particle size of the drug-loaded micelles is increased compared with the blank micelles, while the particle size of the triblock PEG-PLA-SA micelle solution is smaller than that of the diblock PEG-PLA micelle solution. We know that known that both PLA and SA are hydrophobic substances. When these two substances are chemically connected together, they will form a more stable and compact part leading to the formation of slightly smaller particle of the triblock micelle in size as shown in Table 2. As observed by TEM, the shape of the blank and drug-loaded micelles is mostly spherical but displayed smaller in size by DLS. This may be resulted from different detection environments with DLS in solution, while TEM in dry sample.

In order to optimize the drug-loading performance of the triblock polymer, a single factor and response surface experiment was designed. The single factor experiment determined the affecting factors including reaction temperature, reaction time, the molar amount of stearic acid added to the reaction, and L-lactide quality. The dosage of L-lactide directly affects the hydrophobic segment, the reaction temperature and time affect the synthesis efficiency of the triblock polymer, and the amount of stearic acid affects the success rate of the triblock polymer synthesis. Therefore, these four factors have the greatest impact on the encapsulation efficiency of the PEG-PLA-SA triblock polymer. The order of the influence of the four factors on the encapsulation efficiency is the mass of L-lactide>Reaction temperature>Reation time>The amount of SA.

Aspirin reverse drug-loaded micelles were released from the membrane made of PLA, and the drug can be released from the membrane due to the open pores caused by the diffusion or expansion of the polymer material [23]. It can be seen from Figures 9 and 10 that the diblock exhibited a burst of release before 8 h, while the release rate of the triblock was significantly lower than that of the diblock. The release of both triblock and diblock after 8 h tends to be flat. The fast release rate of the diblock and triblock in the first 8 h is due to the large difference in the initial drug concentration, but the reason for slower release of the triblock than the diblock is that the connection of SA affects structural change of the hydrophobic part, resulting in a more stable and compact hydrophobic structure around the triblock polymer, and enhances drug retention ability, leading to decreasing drug release rate. The slow release rate is due to the equilibrium of the drug concentration inside and outside reverse micelles after 8 h. It can be seen from the stability experiment that as the acceleration time increases, the cumulative drug release at the beginning, middle, and end of the month shows a slight increase trend, which may be due to the degradation of PLA dominating the drug release.

## 4. Conclusions

In this study, a PEG-PLA-SA triblock copolymer was synthesized through ring-opening polymerization and esterification. The triblock copolymer self-assembled to form reverse micelles in a toluene/ethanol/water co-solvent system. The obtained reverse micelles were spherical with a particle size between 10 nm-70 nm. Aspirin was successfully added to the hydrophilic core of reverse micelles. In vitro drug release was achieved by embedding the reverse drug-loaded micelles in a biocompatible membrane, in a PH 7.4 PHS buffer solution at 37°C. The triblock copolymer exhibited better drug release potential due to the more stable and compact hydrophobic structure around the triblock copolymer. The response surface experiment took the encapsulation rate as response value and comprehensively explored the cross-effects of these four factors. The best preparation process of PEG-PLA-SA is as follows: reaction temperature at 30.6°C, with 0.036 mol of L-Lactide and 11.6 g of stearic acid, and reaction time of 36.8 h. The predicted value of the encapsulation rate under these conditions was 66.89%. The triblock polymer has good stability of the drug release under accelerated conditions. In summary, the triblock copolymer PEG-PLA-SA exhibits better drug carrier performance than PEG-PLA diblock copolymer in drug loading or drug release. The results indicate that the reverse micelles of PEG-PLA-SA block polymer may be suitable as a continuous drug carrier for aspirin and hydrophilic drugs.
